# Evaluation of Functional Limitations in Female Soccer Players and Their Relationship with Sports Level – A Cross Sectional Study

**DOI:** 10.1371/journal.pone.0066871

**Published:** 2013-06-25

**Authors:** Monika Grygorowicz, Tomasz Piontek, Witold Dudzinski

**Affiliations:** 1 Research and Development Department, Rehasport Clinic, Poznan, Poland; 2 Orthopaedic Department, Rehasport Clinic, Poznan, Poland; 3 Department of Physiotherapy, Institute of Health Protection, Stanislaw Staszic State School of Higher Vocational Education in Pila, Pila, Poland; Universidad Europea de Madrid, Spain

## Abstract

**The main objective(s) of the study:**

The aim of this study was to analyze: a) abnormalities in the length of lower limb muscles, b) the correctness of movement patterns, and c) the impact of functional limitations of muscles on the correctness of fundamental movement patterns in a group of female soccer players, in relation to their skill level.

**Materials and Methods:**

21 female soccer players from Polish Ekstraklasa and 22 players from the 1^st^ Division were tested for lower limb muscle length restrictions and level of fundamental movement skills (with the Fundamental Movement Screen™ test concept by Gray Cook). Chi-square test was used for categorical unrelated variables. Differences between groups in absolute point values were analyzed using the non-parametric Mann-Whitney U test. Statistical significance was set at p<0.05.

**Results:**

Statistically significant higher number of measurements indicating an abnormal length of rectus femoris was observed in the 1st Division group (p = 0.0433). In the group of Ekstraklasa the authors obtained a significantly higher number of abnormal hamstring test results (p = 0.0006). Ekstraklasa players scored higher in the rotational stability test of the trunk (p = 0.0008), whereas the 1st Division players scored higher in the following tests: deep squat (p = 0.0220), in-line lunge (p = 0.0042) and active straight leg raise (p = 0.0125). The results suggest that there are different functional reasons affecting point values obtained in the FMS™ tests in both analyzed groups.

**Conclusions:**

The differences in the flexibility of rectus femoris and hamstring muscle observed between female soccer players with different levels of training, may result from a long-term impact of soccer training on the muscle-tendon system and articular structures. Different causes of abnormalities in fundamental movement patterns in both analyzed groups suggest the need for tailoring prevention programs to the level of sport skills represented by the players.

## Introduction

Soccer is the most popular sport discipline in the world. Statistical data from 2006 demonstrates that the international soccer association, FIFA, unites 207 male and female national federations, and associates over 265 million female and male players. Especially striking is the dynamics of female soccer. In 2006, as compared to 2000, the number of registered female players increased by 54%, reaching 4.1 million (in youth and senior age groups). According to FIFA statistics [1], in 2006 in Poland there were over 180,000 female and 1.8 million male registered players who played soccer professionally and recreationally, and the numbers are still growing.

On the one hand, the growing number of both male and female active players is a positive sign, as it may help raise the skill level in the discipline. However, as the absolute number of players grows, so does the statistical risk of injury [2]. Epidemiological data from a two-year prospective study in the women’s league in the U.S. indicate that the most prevalent injury types include muscle strains (30.7%), sprains (19.1%), contusions (16.2%) and fractures (11.6%), which most often occur in the knee (31.8%), head (10.4%), ankle (9.3%), and foot (9.3%) [3]. Other data indicate that nearly 60% of all injuries are to the lower limbs, and the majority – in the case of non-contact injuries – occur during shots, running or rapid turns [4]. Knee injuries are common and constitute a serious problem regardless of the player’s skill level [5]. They result in long breaks in training, and in some cases they may be the direct cause for leaving the game [6].

Among the internal factors that increase the risk of lower limb injuries in women, as compared to men, researchers most often indicate anatomical considerations [7], hormonal factors (related to changes in the level of sex hormone in different phases of the menstrual cycle) [8], reduced joint congruency (especially in the knee and ankle) [9], biomechanical factors [10], reduced muscle strength (mainly hamstring muscle strength generated in eccentric conditions) [11], impaired muscle balance of flexors and extensors of the knee [9], and different mechanisms of neurological and neuromuscular control [12]. The likelihood of injury is also higher with increased muscle stiffness [13]. According to some authors pain and overload changes are also caused by impaired functional muscle length associated with an increase in passive stiffness [14].

Unfortunately, little is known about the impact of functional muscle restrictions on the level of athletic performance among female soccer players. There are also few papers analyzing the impact of limited flexibility of lower limb muscles and the correctness of movement patterns, particularly in this group of athletes, and especially in relation to the skill level. It seems, therefore, that any work that may extend our knowledge in this area is justified, especially that we need to know as much as possible about the complex determinants of sport injuries to be able to implement preventive measures.

Therefore, this paper analyzes the occurrence of functional limitations among female soccer players in relation to their skill level. The authors assume that more experienced players with a higher level of soccer skills will have a more limited flexibility of lower limb muscles compared to players with a shorter training experience and lower skills. In addition, female players with longer training experience will display greater functional impairments in fundamental movement patterns, and they will more often experience pain in the patellofemoral joint. That is why the purpose of this study was to analyze: a) abnormalities in the length of lower limb muscles and pain of the patellofemoral joint, b) the correctness of movement patterns, and c) the impact of functional limitations of muscles on the correctness of fundamental movement patterns in a group of female soccer players, in relation to their skill level.

## Materials and Methods

The study was conducted on a group of 47 Polish female soccer players with different levels of soccer skills. Players with a higher level of soccer skills and more training experience, who were members of the Polish Ekstraklasa teams (Polish Premier League), were included in the “Ekstraklasa” group. Players with a lower level of soccer skills and shorter training experience, representing the teams of the Polish I Liga (1st Division), were included in the group “1st Division”. Additional inclusion criteria were as follows: participation in training for at least 12 months, no pain in the musculoskeletal system, and no balance disorders on the day of testing. Four players were excluded from the study for health reasons: in soccer players from the 1st Division palpation revealed pain in the lumbar spine (1 case), and persistent knee pain (1 case); two players from the Ekstraklasa group experienced knee pain with knee instability symptoms (positive anterior drawer test). Thus, the Ekstraklasa group comprised 21 players and the 1st Division group –22 players. Descriptive statistics of women from the two research groups are given in Table 1.

**Table 1:** Descriptive characteristics of players participating in the study.

Feature	Group	Median ± SEM	p value of Mann Whitney U Test
Age [yrs ]	Ekstraklasa (n = 21)	23.0±0.760	p<0.0001
	1 st Division (n = 22)	19.0±0.366	
Height [cm ]	Ekstraklasa (n = 21)	164.0±1.506	p = 0.2244
	1 st Division (n = 22)	163.0±1.478	
Weight [kg ]	Ekstraklasa (n = 21)	59.00±1.073	p = 0.3192
	1 st Division (n = 22)	57.25±1.548	
Training experience [yrs ]	Ekstraklasa (n = 21)	9.0±0.736	p<0.0001
	1 st Division (n = 22)	3.5±0.479	

The study was conducted with the approval of the Bioethics Committee at the Karol Marcinkowski Poznan University of Medical Sciences. All soccer players were informed of the purpose and principles of the study, and each gave her written consent to participate in the study. The players were also informed of the right to refuse participation in the study at any time.

Each player was subjected to a physiotherapeutic examination including clinical evaluation and muscle length assessment of the iliopsoas, rectus femoris and hamstring muscle. The mobility of the patellofemoral joint was tested as well. In addition, the level of fundamental movement skills was diagnosed in each subject in accordance with the FMS™ (Fundamental Movement Screen) test concept by Gray Cook. The players completed a questionnaire concerning their training experience, position on the field, and previous injuries (Table 2). None of the players reported any discomfort on the testing day. The researchers began the examination with assessing muscle length and patellofemoral joint pain, and then they conducted fundamental movement tests.

**Table 2:** Number of players reporting injuries.

Area ofinjury	Type of injury	1stDivision	Ekstraklasa
Hip	dysplasia of the hip		1
Knee	medial meniscus injury	1	1
	patellar dislocation	2	
	knee sprain	1	3
	ACL tear		3
	knee cartilage tear		1
	hamstring strain	2	
	quadriceps strain	1	2
	adductor strain		1
Ankle	ankle sprain	8	9
	fracture of the lateral malleolus	1	
	ankle ligament tear		3
	Achilles tendonitis	1	
Other	lumbosacral pain		2
	discopathy of the lumbar spine		1
	overload pain of the spine	1	
	hallux fracture	1	1
	wrist fracture	1	1

### Assessment of Muscle Length and Pain in the Patellofemoral Joint

The length of each muscle was evaluated in an isolated position. Hamstring flexibility was evaluated according to the method of supplementary angle analysis [13]. The methodology described by Witvrouw et al. [15] was used to test the length of the quadriceps. To assess the length of the iliopsoas muscle the authors used the modified Thomas test [16]. The range of motion, level of tension and any subjective sensations of the tested players were compared bilaterally. Diagnostics was conducted during passive stretching of the tested muscles. The test results are presented in a dichotomous scale: normal result (if the test criteria were met) – and abnormal result (if the criteria for performing the test properly were not met, e.g. when the subject felt pain during the test, the heel did not touch the buttock during the quadriceps muscle test, if there was incomplete extension of the knee during the hamstring test, or if the opposite leg was raised during the test of the iliopsoas muscle). Impairment in the patellofemoral joint was assessed using the Clark’s test [17].

### Assessment of the Level of Fundamental Movement Skills

The level of fundamental movement skills was assessed according to the principles described by Gray Cook, the author of the FMS™ method [18], [19]. For each of the soccer players the researchers recorded the points scored during the following tests: deep squat (DS), hurdle step (HS), in-line lunge (ILL), shoulder mobility (SM), active straight leg raise (ASLR), trunk stability push up (TSPU), rotational stability (RS). According to the criteria described in the literature [18], [19] three points were scored for performing a test properly. Two points were scored when any compensation was observed, and one point if the player was not able to properly perform the required movement pattern. Also, if the subject reported pain at any time during the test, she scored zero points for the test. Each of the players performed each test component three times and the best result was recorded. Every participant performed also three clearing exam: Shoulder Mobility Clearing, Trunk Stability Push Up and Rotator Stability Clearing exam (performed at the end of each specific tests) These movements were not scored. They were simply performed to observe a pain response. If pain was produced, a score of zero is given to the entire specific test. All clearing tests were performed in accordance with Cook et al. [18]. The values obtained in each component of the FMS™ test and the composite scores are presented in absolute point values (Table 3 and 4) and in a dichotomous scale (normal vs. abnormal result) (Table 4). The authors decided to use the FMS™ test in this study based on publications which emphasize that the test may be useful in identifying “weak links” in the biokinematic chain, and that it is increasingly often used in the analysis of male and female athletes movement patterns in different sport disciplines [20]. Currently several previous reliability studies for the FMS™ using the standard scoring system have been published [21]–[26]. Overall, inter-rater reliability ranged from poor (κ_a_ = 0.38 [23]), throughout good (ICC = 0.87 and ICC = 0.89 [22]) to high (ICC = 0.92 [24] and κ_w_ = 0.98 [24]), depending on the performed statistical analysis. For intra-rater reliability intra-class correlation coefficients was good and ranged from ICC = 0.6 [23] to ICC = 0.946, respectively to the experience level [26]. Moreover, normative values for in a young, active population were established [27].

**Table 3:** Analysis of differences in FMS™ test results among players with different soccer skill levels.

Feature	Group	Median	Mode	n of mode	p value of Mann Whitney U Test
DS	Ekstraklasa (n = 21)	2	2	19	p = 0.0220
	1 st Division (n = 22)	2,5	multiple	11	
IL	Ekstraklasa (n = 21)	2	2	17	p = 0.0042
	1 st Division (n = 22)	3	3	16	
HS	Ekstraklasa (n = 21)	2	2	14	p = 0.7273
	1 st Division (n = 22)	2	2	17	
ASLR	Ekstraklasa (n = 21)	2	2	14	p = 0.0125
	1 st Division (n = 22)	3	3	17	
TSPU	Ekstraklasa (n = 21)	2	3	8	p = 0.7454
	1 st Division (n = 22)	2	multiple	8	
RS	Ekstraklasa (n = 21)	3	3	13	p = 0.0008
	1 st Division (n = 22)	2	2	18	
SM	Ekstraklasa (n = 21)	3	3	11	p = 0.3922
	1 st Division (n = 22)	2,5	3	11	
Feature	Group	Median ± SEM		p value of Mann Whitney U Test	
TOTAL	Ekstraklasa (n = 21)	16.0±0.460		p = 0.5393	
	1 st Division (n = 22)	15.5±0.583			

**Table 4:** Percentage and absolute number of players who scored the given no. of points in FMS™ tests in the two groups.

FMS™ test	group	normal	abnormal			
		3	2	1	0	Total (2+1+0)
DS	Ekstraklasa	9.52% (2)	0.00% (0)	0.00% (0)	90.48% (19)	90.48% (19)
	1 st Division	50.00% (11)	0.00% (0)	0.00% (0)	50.00% (11)	50.00% (11)
IL	Ekstraklasa	19.05% (4)	0.00% (0)	0.00% (0)	80.95% (17)	80.95% (17)
	1 st Division	72.73% (16)	0.00% (0)	4.55% (1)	22.72% (5)	27.27% (6)
HS	Ekstraklasa	23.81% (5)	4.76% (1)	4.76% (1)	66.67% (14)	76.19% (16)
	1 st Division	22.72% (5)	0.00% (0)	0.00% (0)	77.28% (17)	77.28% (17)
ASLR	Ekstraklasa	38.10% (8)	0.00% (0)	0.00% (0)	66.90% (13)	66.90% (14)
	1 st Division	72.73% (16)	0.00% (0)	0.00% (0)	27.27% (6)	27.27% (5)
TSPU	Ekstraklasa	38.10% (8)	28.57% (6)	0.00% (0)	33.33% (7)	61.90% (13)
	1 st Division	36.36% (8)	36.36% (8)	0.00% (0)	27.27% (6)	63.63% (14)
RS	Ekstraklasa	61.90% (13)	4.76% (1)	0.00% (0)	33.33% (7)	38.09% (8)
	1 st Division	4.55% (1)	9.09% (2)	4.55% (1)	81.81% (18)	95.45% (21)
SM	Ekstraklasa	52.38% (11)	0.00% (0)	0.00% (0)	47.62% (10)	47.62% (10)
	1 st Division	50.00% (11)	13.64% (3)	13.64% (3)	22.72% (5)	50.00% (11)

Percentage values and number of players.

### Statistical Methods

The characteristics of the studied variables are presented with their respective measures of descriptive statistics. The authors conducted a statistical analysis of qualitative variables, such as abnormalities in the lower limb muscle length and in the results obtained in the various components of the FMS™ test. Researches also assessed the impact of functional limitations of muscles on the correctness of fundamental movement patterns in a group of female soccer players, depending on their soccer skill level. There was no normal distribution, and hence quantitative variables (such as age, height etc.) have been presented using the median ± standard error (SEM), and ranks (dummy variables: muscle combined with the tests) have been presented like quantitative variables; additionally, the mode and its size have been given. Dichotomous variables have been given by means of the percentage. Normality of distribution was verified with Shapiro-Wilk test. The results of each component of the FMS™ test have been presented using ranks: the lower the test result, the higher the assigned value. The results of each component and composite scores were compared using the Mann-Whitney U test. The relationships between the muscles and the groups have been presented using the Pearson’s Chi-square test of independence. Differences between groups regarding muscle flexibility and FMS™ test relation were analyzed with Mann-Whitney U test. The results of each component of the FMS™ test have been presented using ranks: the lower the test result, the higher the assigned value. The results of each component and composite scores were compared using the Mann-Whitney U test. Statistical significance was set at p<0.05. Statistical analysis was performed using Statistica v. 7.1 software.

## Results

First, “descriptive” variables of the groups (age, etc.) were compared using the Mann-Whitney U test. It was revealed that Ekstraklasa is statistically different from the 1^st^ Division in terms of age and training experience; the groups are homogenous as regards the height and weight of the subjects.

### Clinical Assessment of Lower Limb Muscle Length and Evaluation of Patellofemoral Joint Pain

In the Ekstraklasa group, 57.14% of all measurements of iliopsoas flexibility revealed limitations in the muscle length (Table 5). Even greater functional limitations were observed for the rectus femoris muscle (59.52%), and the analysis revealed functional limitations of the hamstring in 61.90% of all tested cases. The high percentage (47.62%) of positive tests for patellofemoral joint pain is also alarming. Among the 1st Division players there was a much smaller number of subjects who tested positive for the patellofemoral joint pain; the test was positive only in 25.0% of all cases. The researchers obtained similar values when measuring the length of the hamstring. In 25.0% of all diagnosed cases functional restrictions of the muscle were noted. The results were much worse for the rectus femoris and iliopsoas muscle, where limitations were found in 79.55% and 65.91% of cases, respectively. During the tests of the rectus femoris, the hamstring muscle group and the test for patellofemoral joint pain, the number of normal and abnormal results was linked to the level of soccer training of the subject (Table 5). Statistically significant higher number of measurements indicating an abnormal length of rectus femoris was observed in the 1st Division group (p = 0.0433). On the other hand, in the group of Ekstraklasa soccer players a significantly lower number of normal hamstring test results was obtained (p = 0.0006). The test for patellofemoral joint pain in the 1st Division group came out negative in the majority of cases (p = 0.0290). Measurements of the iliopsoas muscle revealed no statistically significant differences between the frequencies in the compared groups and their respective sport specific skill level.

**Table 5:** Relationships between the player’s level of training and normal and abnormal results recorded during the assessment of muscle length and patellofemoral joint test.

group	abnormal	normal	p value of Chi-Square Test
Rectus femoris			
1st Division	79.55% (35 )	20.45% (9 )	**p = 0.0433**
Ekstraklasa	59.52% (25 )	40.48% (17 )	
Iliopsoas muscle			
1st Division	65.91% (29 )	34.09% (15 )	p = 0.4034
Ekstraklasa	57.14% (24 )	42.86% (18 )	
Hamstring			
1st Division	25.00% (11 )	75.00% (33 )	**p = 0.0006**
Ekstraklasa	61.90% (26 )	38.10% (16 )	
Patellofemoral joint			
1st Division	75.00% (33 )	25.00% (11 )	**p = 0.0290**
Ekstraklasa	54.38% (22 )	47.62% (20 )	

Percentage values and number of players.

### Tests of Fundamental Movement Skills

The Mann-Whitney U test confirmed significant differences in the results obtained in four FMS™ test components by players with different levels of soccer skills (Table 3). Ekstraklasa players scored higher in the rotational stability test of the trunk (RS, p = 0.0008). In turn, the 1st Division players scored higher in the following tests: deep squat (DS, p = 0.0220), in-line lunge (ILL, p = 0.0042) and active straight leg raise (ASLR, p = 0.0125). There were no statistically significant differences between the groups as regards the values of the remaining FMS™ components and the composite test scores (TOTAL). In Table 4 note the results of the hurdle step test (HS), the trunk stability push up test (TSPU), rotational stability test (RS) and shoulder mobility test (SM). Some soccer players scored 0 points in these tests, which means that they reported pain during the tests. The highest percentage of female players with positive pain symptoms was recorded during the lumbar pain test; it reached 28.57% in the Ekstraklasa group and 36.36% in the 1st Division group (Table 4: TSPU test). More than half of Ekstraklasa players scored the maximum number of points only in the two components of the FMS™ tests: the rotational stability test (RS) and the shoulder mobility test (SM): 61.90% and 52.38% of players, respectively. In the 1st Division group at least half of the subjects scored a maximum of 3 points in four FMS™ test components – in deep squat (DS), in-line lunge (ILL), active straight leg raise (ASLR), and shoulder mobility test (SM): 50%, 72.73%, 72,73%, and 50% of players, respectively.

### Relationship between Muscle Flexibility and FMS™ Test Results

To simultaneously compare the relations between muscles and results of the FMS™ tests and between the two tested groups of female soccer players, ranked variables were established using the following scheme: the value of the dummy variable 1 corresponds to the positive muscle flexibility test result and positive result of one FMS™ test component, the value of the dummy variable 3 corresponds to the negative muscle flexibility test and negative positive result of one FMS™ test component, the value of the dummy variable 2 corresponds to other combinations (negative-positive or positive-negative). Variables established in this way were then compared between the groups using the Mann-Whitney U test.

The results suggest that there are different functional reasons affecting point values obtained in the FMS™ tests in both analyzed groups (Table 6). Both for the Ekstraklasa and 1st Division players the researchers noted statistically significant differences between the values obtained in muscle flexibility tests and the level of fundamental movement skills. Subjects from the 1st Division achieved statistically significant better results than players from the Ekstraklasa in DS and ASLR tests due to proper flexibility of hamstring muscle (3 vs, 1 dummy variable, p<0.0001 and p = 0.0003, respectively).

**Table 6:** Group differences between muscle flexibility and FMS™ test results.

Muscle/joint	FMS™ test	Group	Median ± SEM	mod	N of mod	p value of Mann Whitney U Test
Rectus femoris	DS	Ekstraklasa	3±0.092	3	23	p = 0.2315
		1 st Division	2±0.106	Wielokr.	19	
	IL	Ekstraklasa	2±0.091	2	21	**p = 0.0406**
		1 st Division	2±0.105	2	23	
	HS	Ekstraklasa	2±0.107	3	20	p = 0.2041
		1 st Division	3±0.088	3	27	
	ASLR	Ekstraklasa	3±0.132	3	22	p = 0.1159
		1 st Division	2±0.095	2	27	
	PU	Ekstraklasa	2±0.121	3	18	p = 0.2056
		1 st Division	3±0.114	3	26	
	RS	Ekstraklasa	2±0.120	2	17	**p<0.0001**
		1 st Division	3±0.066	3	33	
	SM	Ekstraklasa	2±0.110	2	21	p = 0.1629
		1 st Division	2.5±0.12	3	22	
Iliopsoas muscle	DS	Ekstraklasa	3±0.092	3	22	**p = 0.0311**
		1 st Division	2±0.092	2	27	
	IL	Ekstraklasa	2±0.090	2	22	**p = 0.0052**
		1 st Division	2±0.100	2	25	
	HS	Ekstraklasa	2±0.111	3	20	p = 0.6815
		1 st Division	2±0.088	Wielokr.	21	
	ASLR	Ekstraklasa	3±0.136	3	22	**p = 0.0311**
		1 st Division	2±0.081	2	31	
	PU	Ekstraklasa	2±0.124	3	18	p = 0.6564
		1 st Division	2±0.101	2	21	
	RS	Ekstraklasa	2±0.123	2	16	**p = 0.0003**
		1 st Division	3±0.087	3	29	
	SM	Ekstraklasa	2±0.113	2	20	p = 0.5087
		1 st Division	2±0.112	2	19	
Hamstring	DS	Ekstraklasa	3±0.092	3	24	**p<0.0001**
		1 st Division	2±0.118	1	20	
	IL	Ekstraklasa	2±0.091	Wielokr.	20	**p<0.0001**
		1 st Division	1±0.115	1	28	
	HS	Ekstraklasa	2.5±0.108	3	21	**p = 0.0201**
		1 st Division	2±0.095	2	27	
	ASLR	Ekstraklasa	3±0.133	3	23	**p = 0.0003**
		1 st Division	1±0.128	1	33	
	PU	Ekstraklasa	2±0.122	3	19	**p = 0.0295**
		1 st Division	2±0.087	2	29	
	RS	Ekstraklasa	2±0.123	2	16	p = 0.2614
		1 st Division	2±0.077	2	31	
	SM	Ekstraklasa	2±0.112	2	20	**p = 0.0406**
		1 st Division	2±0.098	2	23	
Patellofemoral joint	DS	Ekstraklasa	2±0.091	Wielokr.	20	p = 0.1736
		1 st Division	2±0.080	2	29	
	IL	Ekstraklasa	2±0.111	3	20	**p = 0.0239**
		1 st Division	2±0.069	2	35	
	HS	Ekstraklasa	2±0.098	2	22	p = 0.1109
		1 st Division	3±0.089	3	25	
	ASLR	Ekstraklasa	2±0.078	2	30	p = 0.0980
		1 st Division	2±0.023	2	43	
	PU	Ekstraklasa	2±0.100	2	24	p = 0.0980
		1 st Division	3±0.109	3	23	
	RS	Ekstraklasa	2±0.095	2	26	**p<0.0001**
		1 st Division	3±0.070	3	31	
	SM	Ekstraklasa	2±0.118	2	18	p = 0.1553
		1 st Division	2±0.103	2	21	

## Discussion

### Abnormalities in Muscle Length and Patellofemoral Joint Pain

Multi-directional movement patterns and phases involving rapid stops, running with changes of direction, which are typical for soccer, require very good physical conditioning. Even in the simplest elements of the soccer technique (e.g. when passing the ball) it is necessary to apply proper strength and balance of the supporting leg to perform an optimal kick of the ball. Players need a wide range of motion in the joints of the pelvic girdle and in the lower limb to perform effective technical actions. Therefore, proper flexibility of the muscle-tendon system is crucial in the preparation of players for full training and match loads [28].The results of this study demonstrate that functional limitations observed in female soccer players depend on their soccer skill level. For rectus femoris, hamstring muscle group and the patellofemoral joint, the number of normal and abnormal results was linked to the level of soccer training of the subject. Among the 1st Division players, who had shorter training experience, the researchers noted a statistically significant higher number of measurements indicating abnormal length of the rectus femoris muscle (p = 0.0433). In the group with a longer training experience (Ekstraklasa) there was a statistically significant higher number of abnormal results of the patellofemoral pain test (p = 0.0290) and hamstring elasticity test (p = 0.0006).

In a study conducted on 17–30 elite soccer teams from Iceland and Norway, Arnason et al. noted as well that the risk of injury in to this muscle group also increases with age/training experience of the players. A multifactorial regression analysis indicated that in addition to age, previous injury of the hamstring muscle group is another factor conducive to injury of the muscle [29]. Increased stiffness of the hamstring can also be caused by an injury of the anterior cruciate ligament [30]. Properly functioning anterior cruciate ligament (ACL) is largely responsible for the stability of the knee, and a damaged ACL is not able to properly fulfil its stabilizing function. In spite of the impaired signalling mechanism, the stabilization of the joint is then taken over by the hamstring [31]. Although during the tests none of the soccer players reported any injuries of the muscle-tendon system, the questionnaires show that some Ekstraklasa players had history of knee ligament damage. Perhaps the statistically significant difference between the analyzed groups in the obtain values, which indicates an impairment of the knee flexor flexibility (Table 5), is a consequence of the changed stability mechanism described above. Reduced flexibility of knee flexors may also be caused by the specifics of the discipline. Repeated short-term, high-intensity efforts (e.g. sprints, running with changes of direction, continuous deceleration and acceleration) may result in increased muscle tension. Long-term soccer training, with strength-speed elements increasing lower limb muscle mass, also leads to increased muscle-tendon stiffness [32]. According to experts, limited muscle elasticity is an important factor leading to injuries, especially muscle strains and overload injuries [33]. Research by Witvrouw et al. also suggests that impaired hamstring flexibility is an intrinsic factor of musculoskeletal injury. Using logistic regression, the researchers found a statistically significant relationship between players with reduced hamstring muscle group flexibility and injuries to the muscle. Similar trends were observed for the quadriceps [15]. Impairments in the flexibility of the muscles leads to changes in their normal functions. Only the correct co-contraction of the antagonists of the knee ensures appropriate muscle balance [34]. Abnormal length-strength characteristics of these muscles can also contribute to increased risk of injury to lower limbs, especially during dynamic movements with a decelerating phase [35].

A high percentage of limited flexibility of the tested muscles, observed in both groups of players, may be alarming. The results indicate significant abnormalities in the functioning of key lower limb muscles in both groups of soccer players. Such overloads usually result in functional compensation in other segments of motion and may be the cause of pain reported by the players. The thesis is corroborated by the percentage values obtained by subjects who reported patellofemoral joint pain (25% and 47.62% of positive tests in 1st Division and Ekstraklasa groups, respectively) or lumbar spine pain (36.36% and 38.10% of positive tests in 1st Division and Ekstraklasa groups, respectively – see Table 4). Due to existing structural changes even minor training loads may pose a serious risk to articular structures, leading to overload and disorders in the proper functioning of joints [36]. Significant stiffness of the hamstring muscle group may be also a factor that determines the risk of injury [37], similarly to the confirmed etiopathogenesis of changes and pain of the patellofemoral joint resulting from repeated training loads and micro-lesions, which are also typical for the discipline [38].

### Proper Performance of Movement Patterns

In the second part of the study the authors analyzed the correctness of fundamental movement patterns, in accordance to the FMS™ concept and according to the represented soccer skill level. The results show there are differences between the scores obtained in some FMS™ test components and the represented level of training. Players with a longer training experience (Ekstraklasa) scored statistically higher in the rotational stability test (RS) than players in the group with a shorter training experience (Table 3). The results can be justified by the specifics of soccer exercise: many technical elements performed on the field requires from players multidimensional actions. For example, to properly change the direction when running, or to dribble, a player needs an appropriate transfer of strength in the entire biokinematic chain: starting with the ground reaction force, through lower limbs, to the trunk and upper limbs. It seems therefore, that repeating technical elements in the axial plane may affect the functional condition of muscles responsible for core stability.

Previous research confirms that the composite score of the FMS™ test less than 14 increases the risk of sustaining an injury during the season from 15% to 51% [39]. The lowest values in the analyzed groups of players from the 1st Division and Ekstraklasa were 10 and 12 points, respectively, which indicates a risk of injury. The most prevalent issues among the subjects included lack of stability in the lumbo-pelvic-hip complex, limited mobility of ankle and hip joints, abnormal axial alignment of lower limbs, and deficits in core and rotational stability. Pain in the lumbar spine had a significant impact on the scores of 14 players. The results reveal the need for implementing specialized correction and prevention training in the areas which constitute the weakest links of the biokinematic chain of lower limbs and trunk – as a necessary extension of soccer training. The results of studies examining professional American football players corroborate the thesis that a focused training program (optimized in each player on the basis of an individual FMS™ assessment) can significantly reduce the percentage of players whose pre-season evaluation results predestine them to injury [40].

### Relationship between Muscle Flexibility, Patellofemoral Joint Pain and FMS™ Test Results

The obtained values of statistical significance between normal and abnormal results of muscle flexibility tests and tests for patellofemoral joint pain, and normal and abnormal FMS™ test results suggest that there are different functional reasons that affect point values scored by players in the FMS™ test components depending on their skill level. Among players with a lower skill level, we observed a relationship between the values obtained in functional tests of the lower limb and the proper flexibility of the hamstring muscle group (Table 6). Higher values obtained in the DS, ILL and ASLR by 1st Division players may result from smaller functional restrictions. Better elongation of the hamstring and less painful patellofemoral joint allow the players to perform deep squat, in-line lung and active straight leg rise movement patterns more correctly. However, in the group of players with a higher skill level, the results were most often affected by the functional length of the iliopsoas muscle, rectus femoris, and hamstring.

Studies show a relationship between impaired muscle flexibility and muscle strength of adductor muscles of the hip [41]–[43]. Numerous papers confirm also the differences in the kinematics of the trunk, and hip and knee joints among male and female soccer players [44]–[49]. However, we found no papers on the impact of muscle flexibility restrictions on fundamental movement skills in female soccer players with different levels of soccer training. Hence it is difficult to compare the results of our study to the results of other authors. The relationships observed in this study may suggest that female soccer players with different training experience require different preventive measures to reduce functional impairments. However, given the small size of the research group, the research should be extended to a larger population of female soccer players to verify this thesis.

### Conclusions

1. The differences in the flexibility of rectus femoris and hamstring muscle and in the functioning of the patellofemoral joint, observed between female soccer players with different levels of training, may result from a long-term impact of soccer training on the muscle-tendon system and articular structures.
2. The results of FMS™ tests show the need for implementing specialized correction and prevention training in the weakest links of the biokinematic chain of lower limbs and trunk – as a necessary extension of soccer training.
3. Different causes of abnormalities in fundamental movement patterns in both analyzed groups suggest the need for tailoring prevention programs to the level of sport skills represented by the players.

### Limitations

The study is cross-sectional and the choice of methodology will inevitably result in a selection error. It is therefore difficult to determine whether functional limitations observed in the analyzed group of players depend only on the represented skill level, or if they are the result of a biased selection of subjects to research groups (selection bias). To avoid this bias and ensure closer sample and population probability we would need a longitudinal cohort study to ensure that the characteristics of the sample selected for testing will not differ from the population in terms of frequency of the analyzed characteristics. A much larger group of soccer players would have to be involved, which may be an organizational challenge and would require a far larger financial effort.
